# Efficient independent planar dose calculation for FFF IMRT QA with a bivariate Gaussian source model

**DOI:** 10.1002/acm2.12056

**Published:** 2017-02-28

**Authors:** Feifei Li, Ji‐Yeon Park, Brendan Barraclough, Bo Lu, Jonathan Li, Chihray Liu, Guanghua Yan

**Affiliations:** ^1^ Department of Radiation Oncology University of Florida Gainesville FL USA; ^2^ Department of Biomedical Engineering University of Florida Gainesville FL USA

**Keywords:** flattening filter free, IMRT QA, independent dose calculation, source model

## Abstract

The aim of this study is to perform a direct comparison of the source model for photon beams with and without flattening filter (FF) and to develop an efficient independent algorithm for planar dose calculation for FF‐free (FFF) intensity‐modulated radiotherapy (IMRT) quality assurance (QA). The source model consisted of a point source modeling the primary photons and extrafocal bivariate Gaussian functions modeling the head scatter, monitor chamber backscatter, and collimator exchange effect. The model parameters were obtained by minimizing the difference between the calculated and measured in‐air output factors (*S*_*c*_). The fluence of IMRT beams was calculated from the source model using a backprojection and integration method. The off‐axis ratio in FFF beams were modeled with a fourth degree polynomial. An analytical kernel consisting of the sum of three Gaussian functions was used to describe the dose deposition process. A convolution‐based method was used to account for the ionization chamber volume averaging effect when commissioning the algorithm. The algorithm was validated by comparing the calculated planar dose distributions of FFF head‐and‐neck IMRT plans with measurements performed with a 2D diode array. Good agreement between the measured and calculated *S*_*c*_ was achieved for both FF beams (<0.25%) and FFF beams (<0.10%). The relative contribution of the head‐scattered photons reduced by 34.7% for 6 MV and 49.3% for 10 MV due to the removal of the FF. Superior agreement between the calculated and measured dose distribution was also achieved for FFF IMRT. In the gamma comparison with a 2%/2 mm criterion, the average passing rate was 96.2 ± 1.9% for 6 MV FFF and 95.5 ± 2.6% for 10 MV FFF. The efficient independent planar dose calculation algorithm is easy to implement and can be valuable in FFF IMRT QA.

## 1 Introduction

Flattening filter‐free (FFF) photon beams have recently gained popularity in intensity‐modulated radiotherapy (IMRT) due to their high dose rate, reduced collimator scatter, reduced head leakage and, consequently, reduced out‐of‐field dose to the patient.^1^, ^2^ Flattening filter (FF) was once considered essential in the linear accelerator (linac) design to achieve uniform dose profiles at certain depths. However, with the advent of advanced optimization techniques and beam shaping devices such as multi‐leaf collimator (MLC),^3^ FF is not necessary in delivering IMRT. The characteristics of FFF beams and their advantages over FF beams have been discussed extensively in the literature.^2^, ^4^, ^5^, ^6^, ^7^ With regard to IMRT, in addition to reduced beam‐on time owing to the combination of increased dose rate and faster MLC moving speed of modern linacs,^2^, ^8^, ^9^ dose reduction outside the treatment volume is mostly noteworthy.^10^, ^11^, ^12^ IMRT has been linked with increased risks of inducing secondary cancers due to its low monitor unit efficiency.^13^, ^14^ Therefore, the dose reduction outside the treatment volume in IMRT with FFF beams is clinically significant, especially for pediatric patients.^15^, ^16^ In the study of pediatric IMRT of intracranial tumors using FFF beams, Cashmore et al. found an average reduction in peripheral doses of 23.7%, 29.9%, 64.9%, and 70.0% to the thyroid, lung, ovaries, and testes, respectively, compared to conventional IMRT with FF beams.^11^

The complex three dimensional (3D) dose distributions of IMRT warrant rigorous pretreatment patient‐specific quality assurance (QA) for safe delivery.^17^, ^18^ The conventional practice calls for IMRT QA to be performed with measurements using detectors or detector arrays inside phantoms, which is usually time consuming and labor intensive. Computer‐based independent dose calculation also proves valuable in validating treatment planning system (TPS) though it cannot replace measurement‐based QA.^19^ Most independent dose calculation algorithms compute two‐dimensional (2D) or 3D dose distributions with high spatial resolution (e.g., 1 mm) within a short amount of time. The computed dose distributions are then compared with the dose distributions computed by the TPS for validation. These independent dose calculation algorithms typically employ a source model which describes the source distribution inside the gantry head.^3^, ^20^, ^21^, ^22^ For high accuracy, the dosimetric details of the beam shaping devices such as the collimator jaws and MLC need to be fully considered when calculating the fluence distribution inside the patient or phantom.^21^, ^23^ To speed up the calculation, the fluence distribution is usually convolved with a dose deposition kernel to obtain the dose distribution. Since independent dose calculation cannot be used as a substitute for measurement‐based IMRT QA,^24^ comprehensive geometric and dosimetric QA of the linac, especially the MLC, needs to be performed rigorously on a regular basis.^25^

The removal of the flattening filter has a few effects on the independent dose calculation algorithms. In a conventional linac, the conical‐shaped FF, which preferentially attenuates the forward‐peaked photon beam at the center, acts as an extra‐focal photon source. It contributes up to 11% of the fluence at the isocenter.^26^ Other extra‐focal sources include the primary collimator, the monitor ionization chamber and the collimator jaws, but their combined contribution to the fluence at the isocenter is only 3%–4%.^27^ Thus, the removal of the flattening filter significantly reduces the head scatter which potentially simplifies the required source model for independent dose calculation. It also reduces the number of photons backscattered into the monitor ionization chamber from the collimator jaws which brings further simplification. Other major effects associated with the removal of FF include changes of the lateral beam profiles (from horn‐shaped to cone‐shaped) and the photon beam spectrum. The latter has profound effect on the depth dose distribution which needs special attention if the independent dose calculation algorithm calculates the full 3D dose distribution.

Independent dose calculation algorithms for conventional IMRT with FF beams have been reported by a number of researchers,^20^, ^22^, ^23^ but few have been reported for IMRT with FFF beams.^28^ Cho et al. reported a multisource model for FFF photon beam dose calculation.^3^ They adapted a previously published analytical three source model^22^ to describe the photon source distribution and aimed to calculate the full 3D dose distributions. Due to the lack of consideration of the MLC details (rounded leaf end, tongue‐and‐groove etc.), the algorithm was only validated with planar dose calculations for open photon beams with irregular shapes. Cashmore et al. adapted a virtual source model previously developed for FFF photon beams and applied the model to calculate the 3D dose distribution for FFF IMRT.^28^ The dose calculation was performed with Monte Carlo simulation and good agreement was observed between calculated and measured dose distributions. However, the computationally intensive and time‐consuming Monte Carlo method may not be ideal for IMRT QA where a planar dose distribution in water phantom needs to be quickly calculated.

The purpose of this work is two‐fold: (a) to develop a common analytical source model for both FF and FFF beams to facilitate a direct comparison; (b) to develop an efficient independent planar dose calculation algorithm to facilitate FFF IMRT QA. The source model is similar to the one proposed by Jiang et al. which explicitly models both the extra‐focal radiation and the monitor chamber backscatter with superior accuracy.^21^ The difference is that, in our model, bivariate Gaussian functions were introduced to implicitly model the monitor chamber backscatter and the collimator exchange effect. The source model was optimized and validated with in‐air output factors (S_c_) measured from a clinical treatment unit (Versa HD, Elekta AB Stockholm, Sweden). A direct comparison of the source models for FF and FFF beams was performed for both 6 MV and 10 MV. A convolution‐based dose calculation algorithm was developed to calculate the planar dose distributions in water. The performance of the algorithm was verified by comparing the computed planar dose distributions with the dose distributions measured with a 2D diode array (MapCHECK 1175, Sun Nuclear Corp., Melbourne, FL) for several head‐and‐neck cases.

## 2 Materials and methods

### 2.A The dose calculation algorithm

#### 2.A.1 Analytical source model

An analytical source model was developed for both FF and FFF beams in this work. The primary photons produced in the linac were modeled with a point source located at the linac target.^20^, ^21^, ^22^ The extra‐focal source distribution was represented by the sum of *N* bivariate Gaussian functions positioned in a plane perpendicular to the beam central axis

(1) $$f\left(x,y\right)=\sum_{i=0}^{N-1}\frac{A_{i}}{2\pi \sigma_{x,i}\sigma_{y,i}}e^{-\left[\frac{x^{2}}{2\sigma_{x,i}^{2}}+\frac{y^{2}}{2\sigma_{y,i}^{2}}\right]},$$

where *A*_*i*_, *σ*_*x,i*_, and *σ*_*y,i*_ are the amplitude, the standard deviation along the *x*‐axis (in the cross‐plane direction), and the standard deviation along the *y*‐axis (in the in‐plane direction) of the *i*th Gaussian respectively. The flattening filter and the primary collimator are the dominant contributors of scattered photons in the FF and FFF mode respectively. Thus, the extra‐focal source plane was positioned at the bottom of the flattening filter for the FF beams and at the bottom of the primary collimator for the FFF beams. An asymmetric bivariate Gaussian function was introduced to account for the collimator exchange effect which refers to the fact that the influence of the upper and lower jaws on *S*_*c*_ is different. Note the Versa HD has only one pair of jaws (X jaws) which define the field size in the *y*‐axis and are located below the MLC. In this context, the MLC is considered as the upper jaws (Y jaws).

The fluence at an arbitrary point can be calculated by integrating the source distribution over the area visible to the point. The visible area is defined by back projecting the edges of the beam shaping devices from the calculation point's view onto the source plane. The back‐projection and integration method for fluence calculation has been extensively discussed in the literature.^3^, ^20^, ^21^, ^22^

#### 2.A.2 Off‐axis ratio

Off‐axis ratio refers to the ratio between the dose at a point away from the central axis and the dose at the central axis of the beam at the same depth. In the FFF mode, dose profiles at all depths exhibit a cone shape which can be modeled with rotationally symmetric off‐axis ratio (OAR). We use a fourth degree polynomial *R*(*x*) to model the OAR,

(2) $$R\left(x\right)=\sum_{i=0}^{4}a_{i}x^{i},$$

where *a*_*i*_ are the coefficients. The fluence calculated using the back‐projection and integration method is modified by multiplying with the OAR to bring it closer to a cone shape.

#### 2.A.3 Dose deposition kernel

Analytical dose calculation algorithms use dose deposition kernels to represent the energy transport and dose deposition of secondary particles originating from the initial interaction point in water.^29^ Realistic dose deposition kernels could be calculated with Monte Carlo simulation. However, to keep the dose calculation algorithm and its commissioning simple yet accurate, we use the following analytical kernel which is the sum of three 2D Gaussian functions

(3) $$k\left(x,y\right)=\sum_{i=0}^{2}\frac{A_{i}}{2\pi \sigma_{i}^{2}}e^{-\left(x^{2}+y^{2}\right)/2\sigma_{i}^{2}},$$

where *A*_*i*_ and *σ*_*i*_ are the amplitude and the standard deviation for the *i*th Gaussian respectively.

#### 2.A.4 MLC modeling

The details of individual MLC leaves need to be modeled for accurate dose calculation for IMRT.^30^, ^31^ The characteristics of a MLC leaf affecting the dose calculation include the interleaf transmission, the tongue‐and‐groove effect, and the transmission through the rounded leaf end. The interleaf transmission factor was measured directly under closed MLC leaves. The Versa HD has a small tongue‐and‐groove gap with a projected width of 0.26 mm at the isocenter.^9^ Its dosimetric influence was modeled with a tongue‐and‐groove transmission factor. The Versa HD uses a rounded leaf end design to ensure consistent beam profile penumbra (distance between 20% and 80% intensity) across the field in the leaf movement direction. The transmission through the first centimeter within the leaf tip gradually declines and was modeled with an exponentially decaying function,

(4) $$L\left(x\right)=e^{-\frac{x^{\alpha }}{\beta }},$$

where *α* and *β* are parameters determining the shape of the function.

### 2.B Commissioning

The model commissioning process determined the parameters for Eqs. (1), (2), (3), (4) using optimization. The parameters for the source model can be optimized with in‐air output factors *S*_*c*_. The *S*_*c*_ of selected symmetrical rectangular fields was measured for this purpose. A cylindrical ion chamber with 3 mm inner radius (IC‐10, Wellhofer Dosimetrie, Germany) was used in a cylindrical mini‐phantom with 4 cm diameter, 10 cm build‐up depth and 5 cm backscatter depth. The measurement for fields with field sizes larger than 6 × 6 cm^2^ was performed at isocenter with a source‐to‐detector distance (SDD) of 100 cm; an extended SSD of 140 cm was used for smaller fields. *S*_*c*_ was also calculated by integrating the source distribution over the area visible to the isocenter. The computation was efficiently performed by taking advantage of the fact that the integration of the Gaussian function could be expressed using the error function. The parameters in Eq. (1) were obtained by minimizing the difference between the measured and calculated *S*_*c*_ of selected fields in a least square sense. The fields selected for commissioning included square fields with field sizes from 3 × 3 to 35 × 35 cm^2^ and rectangular fields with the upper jaws and lower jaws sequentially fixed at 10 cm opening and the other jaws varied from 4 to 30 cm. For validation, *S*_*c*_ for rectangular fields with the upper jaws and lower jaws sequentially fixed at 4 cm opening and the other jaws varied from 4 to 30 cm were used.

The commissioning for the off‐axis ratio, dose deposition kernel and rounded leaf end used a similar approach except the cross‐beam profiles were used. The beam profiles of selected fields were collected with a standard scanning ion chamber (CC13, Scanditronix Wellhofer, Bartlett, TN) in a 48 × 48 × 48 cm^3^ water tank (Wellhofer Dosimetrie, Shwarzenbruck, Germany). All the scans were performed at 10 cm depth with a source‐to‐surface distance of 90 cm. The optimized analytical source model was used to compute beam profiles of the same setting. To this end, the back‐projection and integration method was used to compute the planar fluence, which was subsequently multiplied by the off‐axis ratio, and convolved with the dose deposition kernel to obtain the planar dose distribution. The parameters of Eqs. (2), (3), (4) were sequentially optimized by minimizing the difference between the measured and computed cross‐beam profiles. First, two large fields (30 × 30 cm^2^ and 40 × 40 cm^2^) were used to determine the coefficients of Eq. (2) for the off‐axis ratio modeling. Since the dose deposition kernel was optimized at a later stage, the kernel published in a previous study for FF photon beams with the same nominal energy was used at this step.^20^ The uncertainty was expected to be negligible since only the in‐field, low‐gradient portion (1 cm from the field edges) of the cross‐beam profiles was used to fit the parameters for off‐axis ratio. Second, four fields with field sizes 5 × 5, 10 × 10, 20 × 20, and 30 × 30 cm^2^ were used to optimize the parameters for the dose deposition kernel and the MLC leaf end transmission. The beam profiles in the in‐plane direction (defined by the X jaws) were used for dose deposition kernel optimization to exclude the influence of the rounded leaf end. Finally, with the optimized dose deposition kernel, the beam profiles in the cross‐plane direction (defined by the rounded end of MLC leaves) were used to optimize the parameters for rounded leaf end transmission.

In the commissioning process, the impact of the volume averaging effect (VAE) of the scanning chamber was accounted for using the method of Barraclough et al.^32^ VAE refers to the phenomenon that the measured beam profile is smoothed by the large volume of the ionization chamber which broadens its penumbra. The computed beam profiles were convolved with the detector response function of the CC13 ionization chamber before being compared with the measured beam profiles. The detector response function of CC13 was modeled with a Gaussian function, the parameter of which was determined using the method suggested by Barraclough et al.^32^ The convolution mimicked the VAE associated with the beam profile scanning using the finite‐sized ionization chamber. When the convolved profiles matched the profiles measured with CC13, the computed profiles (before convolution) were expected to match the implicit “true” beam profiles, unperturbed by the VAE.

### 2.C Validation

The performance of the independent planar dose calculation algorithm was further validated using a 2D diode array (MapCHECK 1175, Sun Nuclear Corp., Melbourne, FL). Five head‐and‐neck FFF step‐and‐shoot IMRT plans were generated with the direct machine parameter optimization option in a commercial TPS (Pinnacle^3^, Version 9.8, Philips Radiation Oncology Systems, Fitchburg, WI) for both modalities (6 MV and 10 MV FFF). Each plan consisted of seven beams and no more than 70 segments with 4 cm^2^ minimum segment area. The beams were delivered with 0^o^ gantry angle and measured with MapCHECK. The measurement was performed in a solid water phantom (10 cm buildup). The beam information was also exported from the record‐and‐verify system (MOSAIQ, Elekta Oncology Systems Ltd. Crawley, UK), which was used as input to the proposed algorithm for planar dose distribution calculation. All the calculations were done using a 1.0 × 1.0 mm^2^ dose grid. The measured and calculated planar dose distributions of individual beams were compared using Gamma comparison with percent dose difference (%Diff) and distance‐to‐agreement (DTA) criteria.

Diode detectors are known to exhibit dose rate dependence.^33^ To quantify the dose rate dependence of the MapCHECK diodes, a 20 × 20 cm^2^ open FFF beam with 300 monitor units (MU) was delivered and measured with MapCHECK in the linac service mode with nominal dose rates of 200, 400, 600, 1000, 1400, and 1600 MU/min for 6 MV and 200, 400, 600, 1000, 1400, and 1800 MU/min for 10 MV. The difference in the diode response was evaluated.

## 3 Results

**Figure 1:**
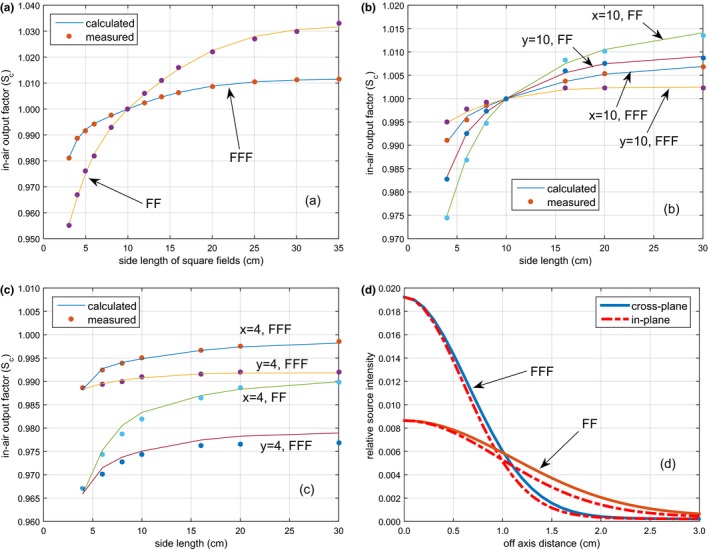
Source model commissioning results for the 6 MV FF and FFF beams. The model parameters were optimized with in‐air output factors (S_c_) of selected square fields (a) and rectangular fields with one pair of jaws fixed at 10 cm (b). S_c_ of rectangular fields with one pair of jaws fixed at 4 cm was used to validate the source model (c). The bivariate Gaussian source distribution is shown in (d).

**Figure 2:**
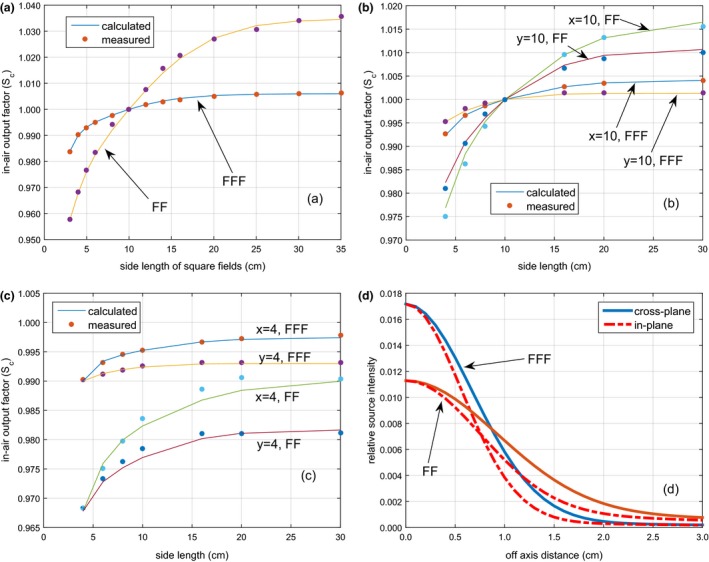
Source model commissioning results, similar to Fig. 1, but for the 10 MV FF and FFF beams.

Figure 1 shows the source model commissioning results for the 6 MV FF and FFF beams. Figures 1(a) and 1(b) show the fitting results with square and rectangular fields with one pair of jaws fixed with 10 cm opening respectively. Figure 1(c) shows the validation results with rectangular fields with one pair of jaw fixed with 4 cm opening. The shape of the bivariate Gaussian distribution is shown in Fig. 1(d). Two bivariate Gaussians are sufficient to achieve good accuracy. For the 6 MV FF fields, the maximum errors were 0.14% in the fitting and 0.20% in the validation; for the 6 MV FFF fields, the maximum errors were 0.08% and 0.03% respectively. The results for the 10 MV FF and FFF beams are shown in Fig. 2. For the 10 MV FF fields, the maximum errors were 0.23% in the fitting and 0.22% in the validation; for the 10 MV FFF fields, the maximum errors were 0.05% and 0.04% respectively. The best‐fit parameters for the source model are listed in Table 1. With the removal of the flattening filter, the intensity of the Gaussian source (relative to the primary source) reduced from 10.48% to 6.84% for 6 MV and from 10.97% to 5.56% for 10 MV. The intensity at the center of the extra‐focal source increased from 0.009 for 6 MV FF to 0.019 for 6 MV FFF and from 0.011 for 10 MV FF to 0.017 for 10 MV FFF, but the average full‐width‐at‐half‐maximum (FWHM) of the source distribution decreased from 1.28 to 0.75 cm for 6 MV and from 1.05 to 0.73 cm for 10 MV.

**Table 1:** The best‐fit parameters for the bivariate Gaussian source model for the FF and FFF beams from an Elekta Versa HD treatment unit

**Beam**	**A_0_**	***σ*** **_x,0_**	***σ***_**y,0**_	**A**_**1**_	***σ***_**x,1**_	***σ***_**y,1**_	**A**_**0**_ **+A**_**1**_
6 MV FF	0.0511	4.2684	3.2619	0.0537	1.0977	0.9659	0.1048
6 MV FFF	0.0220	3.3579	3.3040	0.0465	0.6471	0.6049	0.0684
10 MV FF	0.0648	3.7172	2.7917	0.0449	0.9223	0.7526	0.1097
10 MV FFF	0.0158	2.7368	2.5240	0.0398	0.6671	0.5644	0.0556

**Figure 3:**
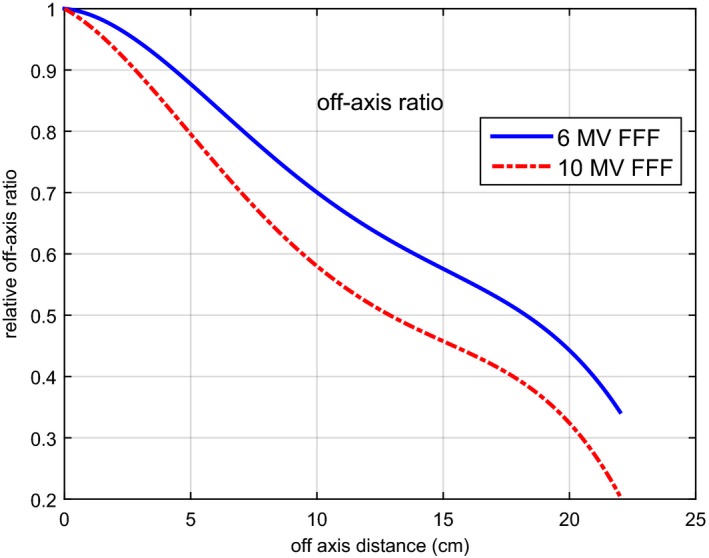
The fitted off‐axis ratio for the 6 MV FFF and 10 MV FFF beams from an Elekta Versa HD treatment.

Figure 3 presents the fitted off‐axis ratio for both 6 MV and 10 MV FFF beams. The coefficients of the fourth degree polynomial are listed in Table 2. The best‐fit parameters for the kernels are listed in Table 3. For the MLC modeling, the interleaf transmission factor and tongue and groove factor were both 0.01. For leaf‐end transmission modeling, the parameters for Eq. (4) were *α* = 0.70 and *β* = 0.24 for both energies.

**Table 2:** The coefficients of the polynomial modeling the off‐axis ratio for the 6 and 10 MV FFF beams from an Elekta Versa HD treatment unit

Energy	a_0_	a_1_	a_2_	a_3_	a_4_
6 MV FFF	5.5049e‐1	−2.1479e‐3	−3.4245e‐3	2.5978e‐4	−6.0768e‐6
10 MV FFF	5.0882e‐1	−1.1488e‐2	−3.0966e‐3	2.8120e‐4	−7.0324e‐6

**Table 3:** The best‐fit parameters of the dose deposition kernel for the 6 and 10 MV FFF beams from an Elekta Versa HD treatment unit

Energy	A_0_	*σ*_0_	A_1_	*σ*_1_	A_2_	*σ*_2_
6 MV FFF	0.8709	0.2241	0.0178	1.4376	0.0032	8.4279
10 MV FFF	1.0385	0.2421	0.0298	1.3446	0.0033	8.3493

**Figure 4:**
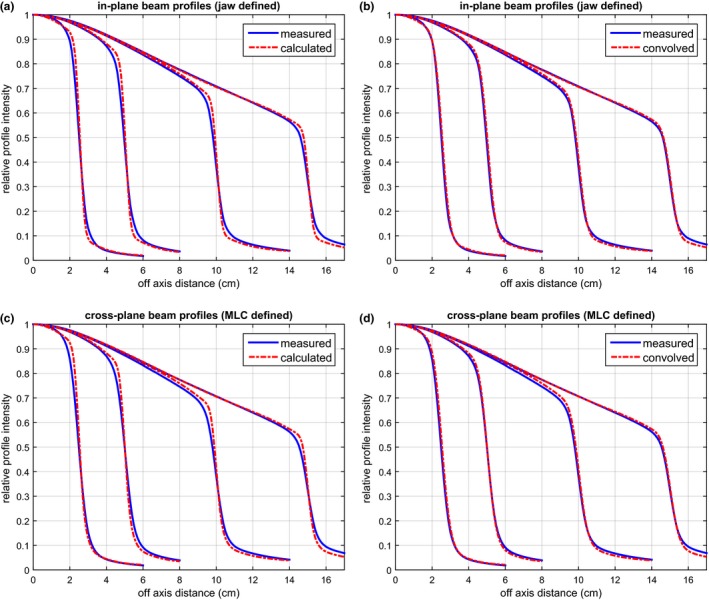
Comparison between the measured and the calculated beam profiles for the 6 MV FFF beams. The comparisons for the in‐plane and cross‐plane beam profiles are shown in (a) & (b) and (c) & (d) respectively. In (a) and (c), the calculated beam profiles exhibit sharper gradient in the penumbra than the measurement due to the use of a deconvolution method to eliminate the volume averaging effect. When convolved with the detector response function, the calculation matches the measurement as shown in (b) and (d).

**Figure 5:**
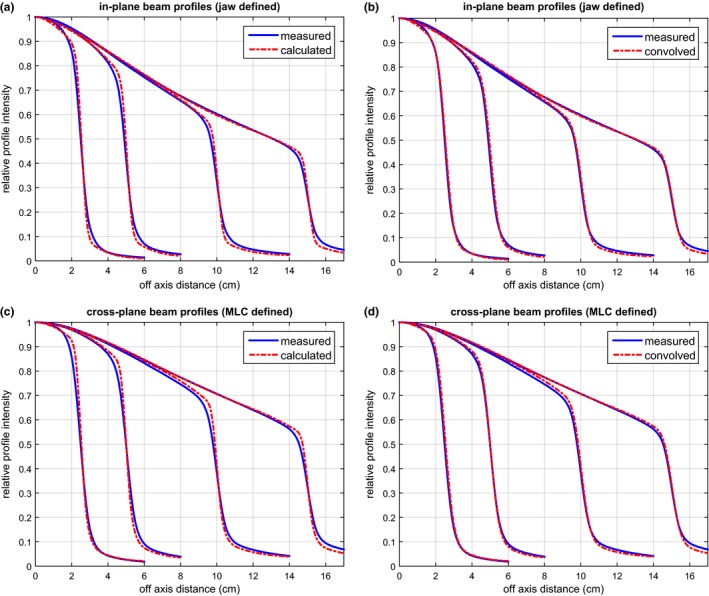
The comparison between the measured and the calculated beam profiles, similar to Fig. 4, but for the 10 MV FFF beams.

Figure 4 shows the comparison between the measured and calculated beam profiles for the 6 MV FFF beam. The comparison for the in‐plane beam profiles (defined by the x jaws) is shown in Figs. 4(a) and 4(b); the comparison for the cross‐plane beam profiles (defined by the MLC leaf ends) is shown in Figs. 4(c) and 4(d). A deconvolution method was used in the commissioning to account for the ionization chamber VAE. Its effectiveness is demonstrated in Figs. 4(a) and 4(c) as the calculated beam profiles exhibit sharper gradient in the penumbra than the measurement. When convolved with the detector response function, the calculated beam profiles closely match the measurement (within 1%/1 mm), as shown in Figs. 4(b) and 4(d). Similar phenomenon can be observed in the comparison for the 10 MV FFF beam as shown in Fig. 5.

The MapCHECK diodes showed similar dose rate dependence for both FFF energies. The response of the diode in the center gradually increased with an increase in the nominal dose rate. When normalized to the response at 600 MU/min, its relative response increased from 0.995 at 200 MU/min to 1.003 at 1600 MU/min for 6 MV FFF and from 0.995 at 200 MU/min to 1.004 at 1800 MU/min for 10 MV FFF. However, when comparing the dose maps measured at different dose rate to that measured at 600 MU/min, the passing rate was all 100% with a 1% dose difference criterion and a 10% threshold for both energies.

**Figure 6:**
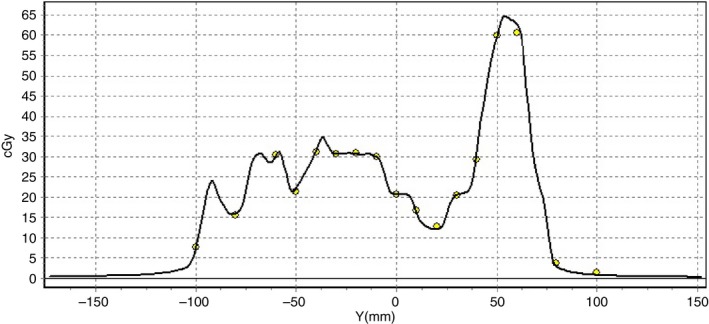
Comparison between the measured (circle) and calculated (line) dose profiles extracted from a 6 MV FFF head‐and‐neck step‐and‐shoot IMRT plan using the MapCHECK software.

For the head‐and‐neck IMRT plans, the average passing rate between the MapCHECK measurement and the calculation was 96.2 ± 1.9% for 6 MV FFF and 95.5 ± 2.6% for 10 MV FFF when the 2%/2 mm criterion with local dose**–**error criterion and the 10% dose threshold was used. When the 3%/3 mm criterion was used, the average passing rate was 99.2 ± 0.9% and 98.9 ± 1.2% for the 6 MV and 10 MV respectively. Figure 6 illustrates the close agreement between the measured and calculated profiles extracted from a typical 6 MV FFF IMRT plan.

## 4 Discussions

As pointed out by several groups, the removal of FF removes the dominant source of head scattered photons, therefore simplifying the head scatter source modeling. There has been no direct comparison of the analytical source models between FF and FFF beams in the literature. In this work, we developed a source model for both modalities and conducted the comparison.

Good agreement was achieved between the calculated and measured *S*_*c*_ for beams with field size ≥3 × 3 cm^2^ for both energies and both modalities. In general, slightly better agreement was achieved with the FFF beams than with the FF beams for both energies, especially for rectangular fields. The *S*_*c*_ for beams with smaller field sizes was measured but not used in the optimization. For example, for the 2 × 2 cm^2^ beam, the measured and calculated *S*_*c*_ was 0.934 and 0.969, respectively, for the 6 MV FFF beam and 0.937 and 0.973, respectively, for the 10 MV FFF beam. For such small fields, the measured *S*_*c*_ being smaller than the calculated *S*_*c*_ can be attributed to the source occlusion effect and the VAE of the used dosimeter (IC‐10), which is why they were excluded in the optimization.

For both energies, the range of *S*_*c*_ reduces from 8% for FF beams to less than 3% for FFF beams between field sizes of 3 × 3 to 35 × 35 cm^2^ which matches well with the results reported for the same type of linac.^5^ Our results show that the relative contribution of the extra‐focal source to *S*_*c*_ at the isocenter was reduced by 34.7% from FF beams to FFF beams for 6 MV and by 49.3% for 10 MV. These findings are in line with the results reported by Dalaryd et al.^34^ In a Monte Carlo study of an Elekta Precise linac, they found that the contribution of scattered photons from the linac head was reduced by 31.7% for 6 MV and 47.6% for 10 MV with the removal of the flattening filter. The 6 MV and 10 MV FF beams have nearly identical spread in *S*_*c*_ (8%), thus the relative contribution of the extra‐focal source is almost the same (10.48% vs. 10.97%). On the other hand, the 6 MV FFF beam has slightly larger spread in *S*_*c*_ than the 10 MV FFF beam (3% vs. 2.3%), which likely explains why the 6 MV FFF beam has slightly higher relative contribution from the extra‐focal source than the 10 MV FFF (6.84% vs. 5.56%).

The extra‐focal source distribution for the FF beams of both energies has a wider core and taller tail than that for the FFF beams, which can be explained by the shape of the *S*_*c*_ curve as well. Unlike *S*_*c*_ of the FFF beams which quickly tapers off beyond 10 × 10 cm^2^, *S*_*c*_ of the FF beams keeps increasing as field size increases. To sustain the continuous increase, a taller and longer tail in the source distribution is necessary for the FF beams.

With the removal of the flattening filter, the collimator exchange effect was reduced from 1.6% for the FF beams to 0.7% for the FFF beams for 6 MV and from 0.8% to 0.4% for 10 MV. There are two factors contributing to the collimator exchange effect. The first factor is related to the locations of the jaws. When the two sets of jaws are open to define the same field size at isocenter, the isocenter sees a larger portion of the extra‐focal source defined by the lower jaws than by the upper jaws. This effect is accounted for when calculating the photon fluence at isocenter using the back‐projection method. The second factor is related to the radiation backscattered into the monitor chamber by the collimator jaws. The amount of the backscattered radiation depends on the surface area of the jaws exposed to the beam. The upper and lower jaws carry different weightings by virtue of their geometric arrangement. Jiang et al. explicitly modeled the effect by assuming a linear relationship between the amount of backscattered radiation and the exposed surface area of both jaws with superior accuracy (0.1%).^21^ Yang et al. ignored the monitor chamber backscatter and also achieved good accuracy (0.3%) for rectangular fields in their three source model.^22^ In this work, 2D bivariate Gaussian functions were introduced to model the collimator exchange effect for the first time. Though the photons produced in the linac theoretically possess a rotationally symmetric distribution, the good agreement between the measured and calculated *S*_*c*_ of both FF and FFF beams justifies the use of the bivariate Gaussian functions. Interestingly, Dalaryd et al. used an asymmetric focal spot (2.6 mm FWHM in the cross‐plane direction and 0.6 mm FWHM in the in‐plane direction) in their Monte Carlo model^34^ which was obtained by fitting the penumbra of measured cross‐plane and in‐plane beam profiles.

A fourth degree polynomial was used to model the OAR of FFF beams. The good agreement between the calculated and measured beam profiles validates its use. The 10 MV FFF beam, with more penetrating and forward‐peaked photons, had smaller OAR than the 6 MV FFF beam. The optimized OARs showed similar trend as the ones reported by Cho et al. for Varian FFF beams.^3^ They used a numerical model with 50 values (0.5 cm apart) to model the OAR of FFF beams. Consequently, a complicated optimization strategy was used to obtain these values. On the contrary, the fourth degree polynomial has only five parameters which were easier to optimize.

Our goal was to develop an efficient and accurate planar dose calculation algorithm for FFF IMRT QA in homogeneous water phantom, not to calculate the full 3D dose distribution. There was no consideration of the actual photon beam spectrum. Instead, a poly‐energetic dose deposition kernel was used to convert the energy fluence to deposited dose. Our results showed that the analytical kernel, consisting of the sum of three Gaussian functions, was sufficient in describing the dose deposition process for planar dose calculation in water. There was also no consideration of kernel tilting or beam hardening.^25^ These simplifications made the algorithm easy to implement without significant accuracy loss. The reduced off‐axis spectrum variation in FFF beams facilitated the use of a single dose deposition kernel across the field.^34^ In the process of optimizing the kernel, a convolution‐based approach was used to eliminate the negative impact of the ionization chamber VAE. Due to the VAE, a typical scanning ionization chamber broadens the penumbra of beam profiles by 2 to 3 mm.^35^ A TPS commissioned with such beam profiles can result in dose calculation which has suboptimal agreement with measurement, especially when performed with diode‐based dosimeters (e.g., MapCHECK).^33^ The effectiveness of the convolution‐based approach accounting for VAE was clearly demonstrated in Figs. 4 and 5, where the calculated beam profiles had sharper penumbra than the measured ones. After being convolved with the detector response function (to simulate the VAE), the penumbra of the calculated beam profiles closely matched the penumbra of the measured ones for both 6 MV and 10 MV. Equally important was the modeling of the MLC dosimetric characteristics, especially the leaf end transmission. We used an exponentially decaying function with only two parameters to model the leaf end transmission. The good accuracy was also illustrated in Figs. 4 and 5 with beam profiles defined by MLC leaf ends.

Due to the simplicity of the source model and the dose calculation algorithm, the commissioning time was less than two minutes on a regular personal computer. The dose calculation algorithm, implemented in MATLAB, took less than eight‐seconds to calculate the planar dose distribution for a typical IMRT beam.

A limitation of the dose calculation algorithm is that a slight underestimation (~1.5%) can be observed just outside the field edge for the 30 × 30 cm^2^ field. However, its impact is expected to be small since this low‐dose area is usually excluded from the comparison due to the use of the dose threshold (e.g., 10%). The MapCHECK diodes showed dose rate dependence of 0.8% for 6 MV FFF and 0.9% for 10 MV FFF. Our observation of FFF step‐and‐shoot IMRT delivery was that the actual dose rate stayed above 600 MU/min where the dose rate dependency was less than 0.4% for both energies. Thus, the dose rate dependence of the MapCHECK was not accounted for in the comparison, and excellent agreement between the calculated and measured dose distribution was still achieved. A very strict criterion (2%/2 mm with local dose**–**error criterion) was used in the evaluation which is critical for error detection.^18^ The dose calculation model can be extended to calculate planar dose distributions at various depths. However, the benefit of the extension in homogenous water phantom is limited. The algorithm uses back‐projection through all beam‐defining devices (jaws and MLC) to calculate fluence, therefore it can be easily extended for linacs with two sets of independent jaws. It is also worth pointing out that the authors have no intention to suggest acceptance criteria for FFF IMRT QA. Instead, an efficient, easy‐to‐implement planar dose calculation algorithm with superior accuracy is provided to facilitate FFF IMRT QA.

## 5 Conclusions

A direct comparison of the analytical source models for FF and FFF beams from an Elekta Versa HD treatment unit was performed in this study. A source model consisting of bivariate Gaussian functions was used and good agreement between the measured and calculated in‐air output factors was achieved for both FF beams (<0.25%) and FFF beams (<0.10%). Due to the removal of the flattening filter, the relative contribution of the head scattered photons reduced by 34.7% for 6 MV and 49.3% for 10 MV. Based on this source model, an efficient and easy‐to‐implement planar dose calculation algorithm with excellent accuracy (>95% average passing rate with 2%/2 mm when compared with MapCHECK measurement) was developed for FFF IMRT QA.

## Conflict of Interest

The authors declare no conflict of interest.
